# *Vibrio vulnificus* VvpE inhibits mucin 2 expression by hypermethylation via lipid raft-mediated ROS signaling in intestinal epithelial cells

**DOI:** 10.1038/cddis.2015.152

**Published:** 2015-06-18

**Authors:** S-J Lee, Y H Jung, S Y Oh, K K Jang, H S Lee, S H Choi, H J Han

**Affiliations:** 1 Department of Veterinary Physiology, College of Veterinary Medicine, Research Institute for Veterinary Science, and BK21 PLUS Creative Veterinary Research Center, Seoul National University, Seoul, South Korea; 2 National Research Laboratory of Molecular Microbiology and Toxicology, Department of Agricultural Biotechnology, and Center for Food Safety and Toxicology, Seoul National University, Seoul, South Korea; 3 Korea Food Research Institute, Seongnam, South Korea

## Abstract

Mucin is an important physical barrier against enteric pathogens. VvpE is an elastase encoded by Gram-negative bacterium *Vibrio vulnificus*; however, the functional role of VvpE in intestinal mucin (Muc) production is yet to be elucidated. The recombinant protein (r) VvpE significantly reduced the level of Muc2 in human mucus-secreting HT29-MTX cells. The repression of Muc2 induced by rVvpE was highly susceptible to the knockdown of intelectin-1b (ITLN) and sequestration of cholesterol by methyl-*β*-cyclodextrin. We found that rVvpE induces the recruitment of NADPH oxidase 2 and neutrophil cytosolic factor 1 into the membrane lipid rafts coupled with ITLN to facilitate the production of reactive oxygen species (ROS). The bacterial signaling of rVvpE through ROS production is uniquely mediated by the phosphorylation of ERK, which was downregulated by the silencing of the *PKCδ*. Moreover, rVvpE induced region-specific methylation in the *Muc2* promoter to promote the transcriptional repression of *Muc2*. In two mouse models of *V. vulnificus* infection, the mutation of the *vvpE* gene from *V. vulnificus* exhibited an increased survival rate and maintained the level of Muc2 expression in intestine. These results demonstrate that VvpE inhibits Muc2 expression by hypermethylation via lipid raft-mediated ROS signaling in the intestinal epithelial cells.

Gastrointestinal mucosal surfaces serve as the first line of defense against many bacterial and viral infections between the epithelium and the luminal content.^1, 2^ Damage and impairment of the mucus layer facilitate the invasion of pathogenic microorganisms and enhance epithelial cell–microbiota interactions to destabilize the homeostasis of the host immune responses.^3^ Mucin (Muc) 2 is the major intestinal *O*-glycosylated protein produced by goblet cells, having an important role as a physiological barrier via mucus formation.^3^ A lack of Muc2 in mice results in severe colitis propagated by an increased bacterial adhesion to the epithelium and intestinal permeability.^4, 5^ Several studies have been conducted to determine the factors that regulate *Muc2* gene expression, including growth factor,^6^ transcription factors,^7^ and the methylation status.^8^ A recent report showed that bacterial pathogens affect a diverse set of epigenetic factors such as DNA methylation and histone modifications to regulate selective activation or silencing of specific host genes.^9^ Specifically, it was proven that there is a close correlation between the silencing of *Muc2* gene expression and its promoter hypermethylation.^10^ On the other hand, intelectin-1b (ITLN) is a lectin that recognizes sugar motifs specific to bacterial cells walls.^11^ ITLN is produced mainly by goblet cells in intestine.^12^ ITLN is located in the brush border membrane, where it serves as a decoy pathogen receptor against microbial infection,^13^ but the functional role of ITLN and its related signaling pathway on *Muc2* gene expression are yet to be elucidated.

*Vibrio (V.) vulnificus* is a Gram-negative food pathogen which causes septicemia, necrotizing wound infections, or gastroenteritis.^14^ The majority of the virulence effect is known to derive from the secreted toxins encoded by cytolytic pore-forming hemolysin (*VvhA*)^14^ and the multifunctional autoprocessing repeats-in-toxin (*MARTXVv*).^14, 15^ VvpE is a homolog of the hemagglutinin/protease, which is suspected to be a major elastase produced by *V. vulnificus*.^16, 17^ Previous work reported that VvpE, a 45-kDa protein, consists of a 35-kDa N-terminal domain for catalytic activity and a 10-kDa domain for its attachment to the substrate.^18^ Although the functions of VvhA and MARTXVv and their roles in pathogenesis have been well studied^14^ owing to their significant fulminating and destructive actions in human tissues, the VvpE appeared to be dispensable for the virulence effect of *V. vulnificus*.^17^ However, it has been reported that the expression of *VvpE* is regulated by the quorum-sensing regulator in promoting the pathogenesis of *V. vulnificus*.^19^ Thus, the functional role of VvpE remains a topic of much debate. In this study, therefore, we investigate the pathogenic mechanism of VvpE and its related signaling pathway in the regulation of the *Muc2* expression.

## Results

### Regulatory effect of VvpE on Muc2 expression

**Figure 1:**
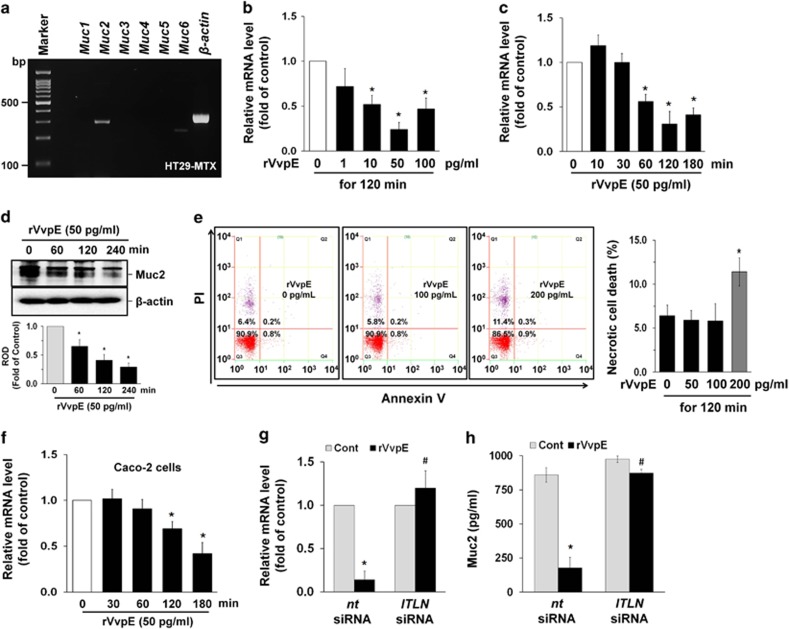
The effect of rVvpE in Muc2 expression. (**a**) Expression of mucin *(Muc)* mRNAs in HT29-MTX cells is shown. *n*=4. Dose (**b**) and time (**c**) responses of rVvpE in *Muc2* expression are shown. *n*=5. *P<0.01 *versus* the cells with no treatment. (**d**) The effect of rVvpE on protein expression of Muc2 was determined by western blot. *n*=4. *P<0.01 *versus* the cells with no treatment. ROD, relative optical density. (**e**) Cells were incubated with 100 and 200 pg/ml of rVvpE for 120 min. Percentages of necrosis, survival, and apoptosis were measured by using PI/Annexin V staining and flow cytometry (left panel). Quantitative analysis of the percentage of necrotic cells (Q1) by FACS analysis is shown (right panel). *n*=5. *P<0.01 *versus* the cells with no treatment. (**f**) The effect of rVvpE on expression of *Muc2* mRNA in Caco-2 cells was determined. *n*=3. *P<0.01 *versus* the cells with no treatment. (**g**) HT29-MTX cells transfected with siRNAs for non-targeting (*nt*) control and *ITLN* were incubated with rVvpE for 120 min. The level of *Muc2* mRNA expression was measured by using real-time PCR. *n*=5. *P<0.01 *versus nt* siRNA+Cont (boiled rVvpE, 50 pg/ml). ^#^*P*<0.05 *versus nt* siRNA+rVvpE. (**h**) HT29-MTX cells transfected with *ITLN* siRNA were incubated with rVvpE for 4 h. The level of Muc2 protein was quantified by ELISA. *n*=5. *P<0.01 *versus nt* siRNA+Cont (boiled rVvpE, 50 pg/ml). ^#^*P*<0.05 *versus nt* siRNA+rVvpE

To find the functional role of VvpE, we used mucus-secreting human HT29-MTX cells, which form a homogeneous population of polarized goblet cells. These types of cells have previously been widely used in the field of mucin-related research.^20^ We first determined the existence of mucin isotypes in HT29-MTX cells. *Muc2* expression, followed by that of *Muc6*, is most abundant in these cells, whereas the expression levels of *Muc1*, *Muc3*, *Muc4*, and *Muc5* were very low (Figure 1a). To evaluate the role of VvpE in the regulation of *Muc2* expression, HT29-MTX cells were exposed to various concentrations (0~100 pg/ml) of rVvpE for 120 min. rVvpE significantly reduced the expression of *Muc2* from 10 to 100 pg/ml compared with the cells with no treatment (Figure 1b). A decrease in *Muc2* mRNA expression and its protein level was observed after 60 min of incubation with 50 pg/ml of rVvpE (Figures 1c and d). Over 200 pg/ml of rVvpE induced a cytotoxic effect by promoting necrotic cell death; however, below 100 pg/ml of rVvpE had an inhibitory effect on *Muc2* expression without cytotoxicity (Figure 1e). In addition, rVvpE (50 pg/ml) also has the ability to inhibit *Muc2* expression in Caco-2 cells (Figure 1f). This result indicates that the functional role of rVvpE is reproducible in other type of human epithelial cells. Moreover, the level of *Muc2* mRNA expression was not changed by treatment with trypsin at 5, 50, and 500 pg/ml for 180 min (Supplementary Figure S1), suggesting that the functional role of rVvpE in Muc2 repression is different from an outcome of general proteolysis of surface proteins. ITLN is thought to confer mucosal protection as a decoy pathogen receptor.^11, 13^ We further investigated the involvement of ITLN in the repression of *Muc*2 elicited by rVvpE (Figure 1g). Interestingly, the inhibitory effect of rVvpE on *Muc*2 expression was silenced by transfection with siRNA for *ITLN*. We also explored the ability of rVvpE to regulate Muc2 production in an enzyme-linked immunosorbent assay (ELISA) assay. In contrast to the control, 50 pg/ml of rVvpE evoked a substantial reduction of Muc2 secretion, whereas the repression activity of rVvpE on Muc2 was blocked by the silencing of *ITLN* (Figure 1h). These results suggest that rVvpE regulates the expression and secretion of Muc2 via ITLN.

### Involvement of a lipid raft and ROS production in *Muc2* regulation

**Figure 2:**
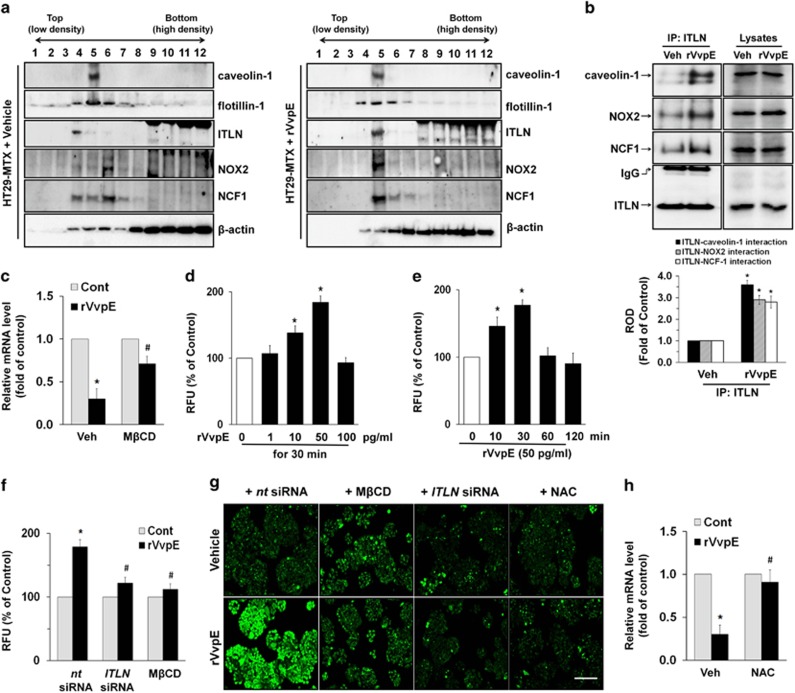
Involvement of a lipid raft and ROS production in *Muc2* regulation. HT29-MTX cells were incubated in the presence of rVvpE (50 pg/ml) for 30 min and then harvested. (**a**) Caveolin-enriched membrane fractions were prepared by discontinuous sucrose density-gradient fractionation, and the location of caveolin-1, flotillin-1, ITLN, and NOX2, and NCF1 was determined by western blot. (**b**) ITLN co-immunoprecipitated with caveolin-1, NOX2, and NCF1 (left side). Expression of caveolin-1, NOX2, NCF1, and ITLN in total cell lysates is shown in the right side. *n*=3. *P<0.05 *versus* Veh (boiled rVvpE, 50 pg/ml). ROD, relative optical density. (**c**) Cells were pretreated with M*β*CD (0.1 mM) for 60 min prior to rVvpE exposure for 120 min. The expression level of *Muc2* mRNA was determined by real-time PCR. *n*=5. *P<0.01 *versus* Cont (boiled rVvpE, 50 pg/ml). ^#^*P*<0.05 *versus* rVvpE alone. Dose (**d**) and time (**e**) responses of rVvpE in ROS production are shown. *n*=5. *P<0.05 *versus* the cells with no treatment. RFU, Relative fluorescence units. (**f**) HT29-MTX cells were pretreated with M*β*CD for 60 min or transfected with *ITLN* siRNA for 24 h prior to rVvpE (50 pg/ml) exposure for 30 min. The level of ROS production is shown. Error bars represent the means±S.E. (*n*=5). *P<0.05 *versus nt* siRNA+Cont (boiled rVvpE, 50 pg/ml). ^#^*P*<0.05 *versus nt* siRNA+rVvpE. (**g**) ROS production (green) was visualized by confocal microscopy. Scale bars, 100 *μ*m. *n*=3. (**h**) Cells were pretreated with NAC (1 mM) for 30 min prior to rVvpE exposure for 120 min. The level of *Muc2* expression is shown. *n*=5. *P<0.01 *versus* Cont (boiled rVvpE, 50 pg/ml). ^#^*P*<0.05 *versus* rVvpE alone

It was previously demonstrated that ITLN is preferentially associated with microvillar lipid rafts.^11, 13^ We further determined the effect of rVvpE on the membrane location of ITLN by means of discontinuous sucrose density-gradient centrifugation. Although caveolin-1 was detected in fraction 5, flotillin-1 was observed in fractions 4–6 (Figure 2a, left panel), indicating that lipid rafts of HT29-MTX cells are presented in fractions 4–6. ITLN at basal condition was distributed in mainly fraction 4, which are correlated with membrane lipid rafts. Interestingly, 50 pg/ml of rVvpE altered the ITLN localization into the fraction 5, despite of the distribution of caveolin-1 and flotillin-1 was not changed (Figure 2a, right panel). Moreover, the subunits of NADPH oxidase (NOX) enzymes, NOX2 (gp91^phox^) and NCF1 (p47^phox^), were highly enriched in the fraction 6. However, rVvpE treatment resulted in translocations of NOX2 and NCF1 into fraction 5 enriched with lipid rafts proteins. Since this approach is qualitative at best, we further tried to quantify the results by using co-immunoprecipitation of ITLN with proteins related to the lipid rafts in the presence of rVvpE. It was noted that ITLN co-immunoprecipitated with caveolin-1, NOX2, and NCF1, and importantly, that these interactions were enhanced by the rVvpE treatment (Figure 2b). These results indicate that rVvpE has ability to cluster ITLN and NOX enzymes without altering their expressions in lipid raft. To confirm the structural importance of membrane rafts in the rVvpE-mediated signaling pathway, we employed the lipid raft sequester methyl-*β*-cyclodextrin (M*β*CD), which is known to deplete cholesterol from the cell membrane. Interestingly, the repression of *Muc2* induced by rVvpE was significantly blocked by a pretreatment with M*β*CD (Figure 2c). Since the tyrosine kinases are closely associated with the lipid raft, we further have confirmed whether rVvpE regulates activation of c-Src and FAK, as representative tyrosine kinases. rVvpE markedly increased the phosphorylation of c-Src and FAK from 10 min (Supplementary Figure S2a). Interestingly, pre-treatment with M*β*CD blocked the c-Src and FAK activation induced by rVvpE (Supplementary Figure S2b). These mean that rVvpE regulates the lipid raft-mediated signaling pathway and thereby modifies the phosphotyrosination of c-Src and FAK and the localization of NOX. Clustering of NOX enzymes facilitates the production of reactive oxygen species (ROS), which results in a prominent amplification of the transmembrane signal.^21^ A significant increase in the ROS level appeared after incubation with 10–50 pg/ml for 30 min compared with the vehicle alone (Figure 2d). The increase in ROS production was transiently augmented between 10 and 30 min after incubation with 50 pg/ml of rVvpE (Figure 2e). In addition, a pretreatment with M*β*CD as well as silencing of *ITLN* significantly blocked rVvpE-induced ROS production (Figure 2f). The regulatory effects of M*β*CD, *ITLN* siRNA, and antioxidant, *N*-acetylcysteine (NAC) on ROS production were further visualized by staining HT29-MTX cells with a fluorescent dye, 2′, 7′-dichlorofluorescein diacetate (CM-H_2_DCFDA) (Figure 2g). Consistently, the repression of *Muc2* by rVvpE was significantly blocked by the treatment with NAC (Figure 2h). These data suggest an involvement of a lipid raft and ROS production in rVvpE-mediated *Muc2* regulation.

### Essential role of protein kinase C (PKC) in *Muc2* regulation

**Figure 3:**
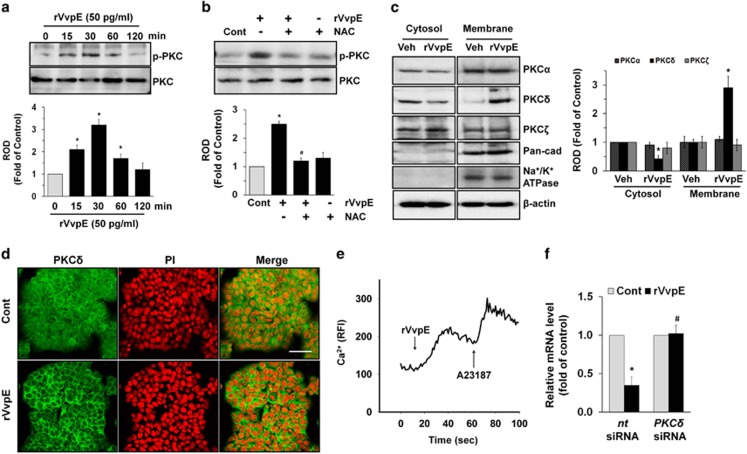
Essential role of PKC in Muc2 regulation. (**a**) Phosphorylation of PKC in cells treated with rVvpE is shown. *n*=3. *P<0.01 *versus* the cells with no treatment. ROD, Relative optical density. (**b**) Cells were pretreated with NAC (1 mM) prior to rVvpE exposure for 30 min. Phosphorylation of PKC is shown. *n*=4. *P<0.01 *versus* Cont (boiled rVvpE, 50 pg/ml). ^#^*P*<0.05 *versus* rVvpE alone. (**c**) Membrane translocation of PKC isoforms in cells treated with rVvpE for 30 min was determined by western blot. Pan-cadherin and Na^+^/K^+^ ATPase were used as a plasma membrane control. *n*=3. *P<0.05 *versus* Veh (boiled rVvpE, 50 pg/ml). (**d**) Membrane translocation of PKC*δ* (green) was determined by confocal microscopy. PI was used for nuclear counterstaining (red). Scale bars, 100 *μ*m. *n*=3. (**e**) Changes in [Ca^2+^]i were monitored by confocal microscopy, and data are expressed as relative fluorescence intensity (RFI, F/F0%, arbitrary unit). A23187 (10 *μ*M) was used as a positive control. *n*=4. (**f**) Cells were transfected with *PKCδ* siRNA for 24 h prior to rVvpE (50 pg/ml) exposure for 120 min. The level of *Muc2* mRNA expression is shown. *n*=4. *P<0.01 *versus nt* siRNA+Cont (boiled rVvpE, 50 pg/ml). ^#^*P*<0.05 *versus nt* siRNA+rVvpE

Given that ROS has an important role as signal messengers in regulating cellular functions through activation of protein kinase C (PKC),^22^ we examined whether rVvpE induces the phosphorylation and translocation of PKC as an important downstream intermediate of ROS in mucus-secreting HT29-MTX cells. rVvpE significantly induced PKC phosphorylation from 30 min (Figure 3a). The phosphorylation of PKC by rVvpE was blocked by NAC (Figure 3b), indicating that the functional importance of ROS in this regulation. In an experiment to identify the specific PKC isotypes, the translocation of PKC*δ*, but not PKC*α* or PKC*ζ*, from the cytosol to the membrane compartment was observed after cells were treated with rVvpE (Figure 3c). The membrane translocation of PKC*δ* was further confirmed by immunofluorescence staining in rVvpE-treated HT29-MTX cells (Figure 3d). We also assessed the involvement of calcium influx during the repression of *Muc2* induced by rVvpE. As shown in Figure 3e, 50 pg/ml of rVvpE induced an increase in calcium influx. A Ca^2+^ ionophore A23187, which increases [Ca^2+^]i, was used as a positive control to validate the results. Importantly, the rVvpE-induced *Muc2* repression was inhibited by transfection with *PKCδ* siRNA (Figure 3f). These data suggest a functional role of PKC*δ* in regulating rVvpE-mediated *Muc2* repression.

### Regulatory effect of VvpE on MAPK activation and *Muc2* promoter methylation

**Figure 4:**
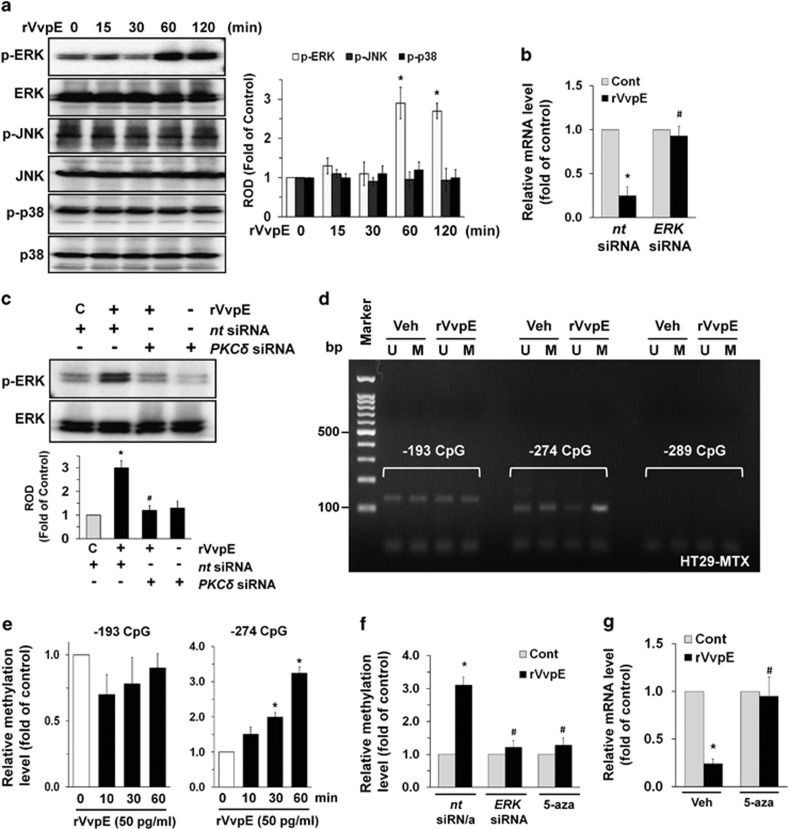
Regulatory effect of VvpE on MAPK activation and *Muc2* promoter methylation. (**a**) The effect of rVvpE on the expression of MAPK was determined by western blot. *n*=4. *P<0.01 *versus* the cells with no treatment. ROD, relative optical density. (**b**) Cells were transfected with *ERK* siRNA for 24 h prior to rVvpE exposure for 120 min. The level of *Muc2* mRNA expression was measured by using real-time PCR. *n*=4. *P<0.01 *versus* Cont (boiled rVvpE, 50 pg/ml). ^#^*P*<0.05 *versus* rVvpE alone. (**c**) Cells were transfected with *PKCδ* siRNA for 24 h prior to rVvpE exposure for 60 min. *n* =3. *P<0.01 *versus nt* siRNA+C (boiled rVvpE, 50 pg/ml). ^#^*P*<0.05 *versus nt* siRNA+rVvpE. C, Control. ROD, relative optical density. (**d**) Genomic DNA treated with rVvpE for 60 min was prepared. Changes in the promoter methylation status of *Muc2* gene was determined by methyl-specific PCR analysis. *n*=4. U, unmethylated form; M, methylated form. (**e**) Time responses of rVvpE in *Muc2* methylation status at −274 and −193 CpG sites are shown. The relative level of *Muc2* methylation is shown, compared to the unmethylated form. *n*=4. *P<0.05 *versus* the cells with no treatment. (**f**) Cells were transfected with *ERK* siRNA for 24 h or pretreated with 5-azacytidine (5-aza) (1 *μ*M) for 30 min prior to rVvpE exposure for 60 min. The relative level of *Muc2* methylation is shown, compared to the unmethylated form. *n*=4. *P<0.05 *versus nt* siRNA+Cont (boiled rVvpE, 50 pg/ml). ^#^*P*<0.05 *versus nt* siRNA+rVvpE. (**g**) Cells were pretreated with 5-aza for 30 min prior to rVvpE exposure for 120 min. The level of *Muc2* mRNA expression was measured by using real-time PCR. *n*=4. *P<0.01 *versus* Cont (boiled rVvpE, 50 pg/ml). ^#^*P*<0.05 *versus* rVvpE alone

We then determined how rVvpE links to the activation of MAPKs, which are interesting candidates as downstream mediators of ROS and PKC in the regulation of mucin production.^23^ rVvpE increased the phosphorylation of ERK between 60 and 120 min (Figure 4a) but did not affect the phosphorylation of JNK or p38 MAPK. Knockdown of ERK1/2 with specific *ERK1/2* siRNA significantly blocked the *Muc2* reduction induced by rVvpE (Figure 4b). In addition, the phosphorylation of ERK evoked by a treatment with rVvpE was markedly inhibited by transfection with *PKCδ* siRNA (Figure 4c). These data represent important evidence that ERK phosphorylation is regulated by the activation of PKC, as required for *Muc2* reduction. Because ERK is known to affect gene methylation directly for epigenetic alteration,^24^ the methylation status following the treatment with rVvpE was determined by means of methyl-specific PCR. A bisulfite treatment converted cytosine residues in the genomic DNA to uracil, which were amplified as thymine during the subsequent PCR. We attempted to analyze the methylation status of the *Muc2* gene promoter containing three CpG sites (−193, −274, and −289), which are critical CpG sites responsible for the repression of *Muc2* expression.^25^ As shown in Figure 4d, primers for −193 and −274 CpG sites that specifically amplified either the methylated or unmethylated form of the *Muc2* promoter produced 137 bp and 105 bp methylated bands from vehicle-treated HT29-MTX cells, respectively, indicating that the *Muc2* promoter was methylated in these alleles. However, the methylated bands of the *Muc2* gene did not appear at the −289 CpG site. Interestingly, 50 pg/ml of rVvpE markedly increased the level of *Muc2* promoter methylation at the −274 CpG site, but not at −193 for 60 min. We further explored the ability of 50 pg/ml of rVvpE to induce methylation for 60 min and quantified the relative value of CpG methylation compared with an unmethylated form in the *Muc2* promoter using a real-time PCR analysis (Figure 4e). The relative level of *Muc2* promoter methylation at the –274 CpG site was significantly augmented by a treatment with rVvpE between 30 and 60 min compared with a control. As expected, the methylation status of the *Muc2* promoter containing the –193 CpG site was not changed by a treatment with rVvpE. Interestingly, the level of *Muc2* promoter methylation at the –274 CpG site was significantly inhibited by knockdown of *ERK1/2* and by treatment with DNA methylation inhibitor 5-azacytidine (5-aza) (Figure 4f). In addition, the pretreatment with 5-aza significantly blocked the repression of *Muc2* as induced by rVvpE (Figure 4g). These data indicate that the ERK-mediated hypermethylation of the *Muc2* promoter is responsible for the low level of *Muc2* expression in rVvpE-treated HT29-MTX cells.

**Figure 5:**
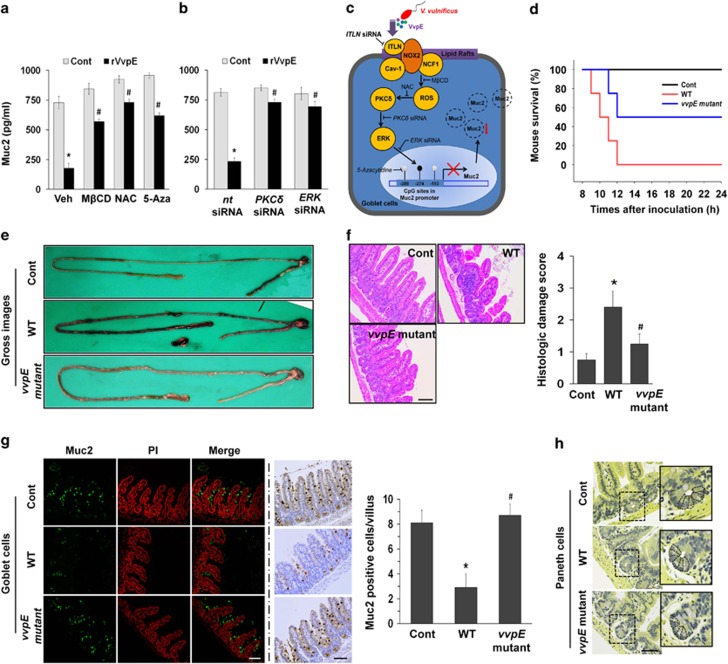
Role of VvpE and its related signal molecules on Muc2 production. (**a**) HT29-MTX cells were pretreated with M*β*CD, NAC, and 5-azacytidine prior to rVvpE (50 pg/ml) exposure for 4 h. The level of Muc2 protein was quantified by ELISA. Error bars represent the means±S.E. (*n*=5). *P<0.01 *versus* Cont (boiled rVvpE, 50 pg/ml). ^#^*P*<0.05 *versus* rVvpE alone. (**b**) Cells were transfected with *PKCδ* siRNA and *ERK* siRNA prior to rVvpE (50 pg/ml) exposure for 4 h. *P<0.01 *versus nt* siRNA+Cont (boiled rVvpE, 50 pg/ml). ^#^*P*<0.05 *versus nt* siRNA+rVvpE alone. (**c**) A proposed model for VvpE-evoked signaling pathway in intestinal epithelial cells. VvpE has an important role in the targeting of *V. vulnificus* for goblet cells, where VvpE represses *Muc2* expression via the regulation of ITLN-mediated ROS production and distinctive hypermethylation of *Muc2* promoter. (**d**) Iron-overloaded ICR mice were received i.p. inoculation of WT, boiled WT (Cont), and *vvpE* mutant at 1.2 × 10^2^ CFU/ml for 24 h. Survival rate of mice infected with *vvpE* mutant relative to WT is shown. *n*=10. *P*<0.05. Mice were given i.g. inoculation of WT and *vvpE* mutant at 1.1 × 10^9^ CFU/ml and killed at 16 h later. (**e**) Gross morphologies of intestines are shown. (**f**) Representative ileum tissues stained with hematoxylin and eosin are shown (left panel). *n*=5. Scale bars represent 100 *μ*m. Average scores of histopathologic damage index from mouse ileum is shown (right panel). *n*=5. *P<0.01 *versus* Cont. ^#^*P*<0.05 *versus* WT. (**g**) Expression of Muc2 in mouse ileum was examined by immunofluorescence (left panel, green) and immunohistochemical analysis (right panel, brown). PI (red) was used for nuclear counterstaining for immunofluorescence analysis. Scale bars represent 100 *μ*m. The mean numbers of Muc2-labeled cells per villi are shown in the bar graph. *P<0.01 *versus* Cont. ^#^*P*<0.01 *versus* WT. Error bars represent the means±S.E. from five number of villi and crypts from five different mice. (**h**) Paneth cells were stained using the phloxine-tartrazine staining method. There were no changes in the mean number of paneth cells per crypt (*n*=5). Scale bars, 50 *μ*m

To ensure the functional role of rVvpE and its related signaling molecules in regulation of transcriptional repression of *Muc2*, we further determined the effect of siRNAs and inhibitors on the activation of the signaling pathway induced by rVvpE using an ELISA assay (Figures 5a and b). We pretreated cells with M*β*CD and NAC to prove the involvement of lipid rafts-dependent ROS production, *PKCδ* and *ERK1/2* siRNAs to confirm involvement PKC*δ* and ERK activation, and 5-aza to show involvement of *Muc2* promoter methylation. As expected, the repression of Muc2 production induced by rVvpE was significantly abrogated by the aforementioned siRNAs and inhibitors for lipid rafts, ROS, PKC, ERK, and methylation, respectively. These results demonstrate the relevance of lipid rafts-mediated ROS signaling pathway and hypermethylation in promoting rVvpE-mediated Muc2 repression. The sequences of putative bacterial signaling pathways of VvpE are summarized in Figure 5c.

To evaluate the clinical relevance of *V. vulnificus* VvpE, we evaluated the effect of VvpE on mouse lethality. We used an iron-overloaded mouse model to bring the growth of *V. vulnificus* within the lethal level.^26^ Seven-week-old iron-overloaded ICR mice inoculated intraperitoneally (i.p.) with boiled *V. vulnificus* (Cont), *V. vulnificus* (WT), and a mutant deficient in *vvpE* gene in *V. vulnificus* (*vvpE* mutant) at 1.2 × 10^2^ CFU/ml for 24 h, after which their survival rates were monitored (Figure 5d). All of the mice injected with WT were dead by 12 h post injection, while 5 out of the 10 mice injected with the *vvpE* mutant remained alive 24 h after i.p. inoculation, thus showing some degree of the attenuation of mouse lethality. These results indicate that VvpE makes an important contribution to the lethality of mice infected with WT. Finally, we further investigated the effect of VvpE on the expression of intestinal Muc2 in mouse. Mice inoculated intragastrically (i.g.) with Cont, WT, and *vvpE* mutant at 1.1 × 10^9^ CFU/ml for 16 h. WT caused severe necrotizing enteritis of the intestine (Figure 5e), where it induced shortened villi heights accompanied by an expanded width and increased inflammation at 16 h infection, resulting in increased level of histopathological damage score, compared with control mice (Figure 5f). However, unlike WT, the mice infected with *vvpE* mutant almost completely maintained their intestinal villi structures, and the inflammation was silenced. The result of the immunofluorescence staining of Muc2 revealed that mice infected with WT for 16 h have fewer goblet cells expressing Muc2, which was negated by infection with *vvpE* mutant (Figure 5g). However, WT did not alter the number of paneth cells in the small intestine for 16 h infection (Figure 5h). Thus, these results indicate that VvpE has an important role in repression of Muc2 during *V. vulnificus* infection.

## Discussion

Our data demonstrate that *V. vulnificus* VvpE is the functional elastase responsible for Muc2 repression in the mouse and that VvpE, in acting through lipid raft-associated ITLN, inhibits *Muc2* expression by stimulating the methylation of the *Muc2* gene promoter through the ROS-dependent activation of PKC*δ*/ERK pathway in mucus-secreting HT29-MTX cells. Many enteric pathogens directly degrade intestinal mucin by producing Pic,^27^ StcE,^28^ or Hap.^29^ However, our result in the present study suggests that *V. vulnificus* uniquely regulates intestinal Muc2 expression by producing VvpE with modes of action that differ from those of other enteric pathogens. It is not clear whether the functional role of VvpE in the repression of Muc2 *in vivo* is a sequential result of the activation of different types of cells in mouse intestine or, alternatively, an independent process involving other cellular signaling events. In addition, it is also possible that the VvpE would impact on the delivery of major toxins (e.g., VvhA, MARTXVv) during intestinal infection of *V. vulnificus*. However, it is clearly reported that the impairment of the mucus layer enhances the colonization of pathogens with establishment of the appropriate portal of entry, where it gains access more easily to epithelial cells and thereby promotes pathogen–host adherence mechanisms. Thus, these results suggest VvpE has important role in the pathogenesis of *V. vulnificus* in intestine. Although previous research has raised some serious doubts about the critical role of VvpE in mouse lethality,^17^ our *in vivo* data revealed that VvpE is a necessary virulence factor responsible the mouse lethality of *V. vulnificus*. This means that the discrepancy with regard to the functional role of VvpE could be because of differences in the pathogenic isolate of *V. vulnificus* (MO6-24/O), the anesthesia method, or the injection location used in this study.^30, 31^ In addition, many relevant reports have suggested that VvpE causes tissue necrosis and increased vascular permeability, which are necessary for the invasion of this bacterium.^32, 33^ Thus, we suggest that VvpE is another important virulence factor of *V. vulnificus* responsible for mouse lethality and Muc2 repression.

Concerning the pathogenic mechanism of VvpE, we showed that a unique relationship between lipid raft-dependent ITLN recruitment and ROS production in the regulation of *Muc2* expression. Our finding that ITLN is localized on lipid raft areas exposed on the membrane surface of mucus-secreting HT29-MTX cells is consistent with a previous report that ITLN was found in intestinal microvillar rafts, enriched in glycoproteins and glycolipids, where it provides a number of carbohydrate-binding sites for microbial adhesion.^13^ However, ITLNs are proposed to serve as a host defense lectin to assist with the phagocytic clearance of microorganisms.^34^ These differ from our data that ITLN is a functional signaling molecule which mediates the virulence effect of VvpE to repress *Muc2* expression. Moreover, previous work revealed that transgenic mice overexpressing ITLN did not show enhanced pathogen clearance,^35^ suggesting that ITLN has a critical role as host mediator of rVvpE in lipid rafts. The important finding of this study is that rVvpE triggers clustering of ITLN and NOX in the lipid raft for the ROS production, which results in a repression of Muc2. Increasing evidence has suggested that lipid rafts are clustered to form a redox signaling platform through gp91^phox^ (NOX2) coupling with cytosolic factors that include p47^phox^ (NCF1), p67^phox^ (NCF1), and small GTPase Rac1, and that these processes subsequently produce superoxides and other ROS.^36, 37^ These results are further supported by a previous study in which several enteric toxins that interact with lipid rafts, including *Helicobacter (H.) pylori* vacuolating toxin^38, 39^ and *Clostridium(C.) perfringens* enterotoxin,^40^ have shown the ability to regulate NOX aggregation via lipid raft clustering as an initial attachment platform, thus having a virulence effect on intestinal pathogenesis.

An influx of Ca^2+^ on PKC-mediated MAPK activation is linked to bacterial stratagems to modulate the host signaling pathway. *H. pylori* and *C. perfringens* have been shown to elicit Ca^2+^ response in the gut.^41^ Moreover, multiple signaling processes, such as those acting through the Ca^2+^ and PKC/MAPK pathways, were rapidly activated in target cells through ROS production.^42, 43^ These previous results are consistent with our current finding that rVvpE induces an influx of Ca^2+^ on PKC/MAPK activation, in that PKC is required for ROS production to repress *Muc2* expression. Indeed, ITLN was shown to evoke Ca^2+^ signaling to recognize the pathogen infection.^11^ Interestingly, rVvpE uniquely regulated *Muc2* repression through two distinct proteins, PKC*δ* and ERK. A previous report showed *H. pylori* activates novel PKC*δ* to control MAPK pathways.^44^ However, other authors showed that the *H. pylori* infection regulated p38 MAPK activation in promoting ROS signaling pathway.^45^ These differ from our data that rVvpE increases ERK phosphorylation via the activation of PKC*δ* during ROS production. On the other hand, a previous report showed conventional PKC*α* activation was found to require for the impairing intestinal barrier function induced by enteropathogenic *Escherichia coli* infection,^46^ suggesting that the cellular pathways differ in different bacterial pathogens type. These data indicate that rVvpE selectively regulates specific PKC isozymes and MAPK phosphorylation. In keeping with the unique signaling pathway of rVvpE repressing *Muc2* expression, our study showed that rVvpE induced the region-specific methylation of the *Muc2* promoter at the −274 site through ERK and that the inhibition of DNA methylation blocks rVvpE-induced *Muc2* reduction. Promoter methylation of cytosine residues at CpG dinucleotides is an important epigenetic mechanism in promoting the transcriptional repression of *Muc2*.^10^ Compelling evidence supports the role of PKC*δ* and ERK in the hypermethylation of tumor-suppressor genes and the pathogenesis of colon cancer.^24^ These data provide the first evidence that bacterial pathogen promotes hypermethylation of the *Muc2* promoter to repress *Muc2* expression.

In conclusion, our results suggest that VvpE regulates DNA methylation via the activation of lipid rafts-mediated ROS signaling pathway to promote the transcriptional repression of *Muc2*. Thus, highlighting the new pathogenic signaling pathways involved in VvpE-induced Muc2 repression may provide potential therapeutic strategies for *V. vulnificus* infections in intestine.

## Materials and Methods

### Chemicals

Fetal bovine serum (FBS) was purchased from BioWhittaker (Walkersville, MO, USA). The following antibodies were purchased: p-PKC and PKC antibodies (Cell Signaling Technology, Danvers, MA, USA); NOX2 antibody (BD Biosciences, Franklin Lakes, NJ, USA); NCF1 antibody (LifeSpan Biosciences, Seattle, WA, USA); ITLN and Na^+^/K^+^ ATPase antibodies (abcam, Cambridge, MA, USA); p-ERK1/2, ERK, p-JNK, JNK, p-p38 MAPK, p38 MAPK, p-*c*-Src, *c*-Src, p-FAK, FAK, pan-cadherin, PKC*α*, PKC*δ*, and PKC*ζ* antibodies (Santa Cruz Biotechnology, Paso Robles, CA, USA); Horseradish peroxidase (HRP)-conjugated goat anti-rabbit and goat anti-mouse IgG antibodies (Jackson Immunoresearch, West Grove, PA, USA); 2′, 7′-dichlorofluorescein diacetate (CM-H_2_DCFDA) was obtained from Invitrogen (Carlsbad, CA, USA). A23187, methyl-*β*-cyclodextrin (M*β*CD), *N-acetyl-l-cysteine* (NAC) and 5-azacytidine were purchased from Sigma Chemical Company (St. Louis, MO, USA). The concentrations of all of pharmacological inhibitors listed did not show any significant cytotoxic effects by themselves as confirmed by FACS analysis in each experiment. All other reagents were of the highest purity commercially available and were used as received.

### Cells

Mucus-secreting human intestinal epithelial (HT29-MTX) and human colorectal epithelial (Caco-2) cells were purchased from American Type Culture Collection (ATCC, Manassas, VA, USA). HT-29-MTX and Caco-2 cells were grown at 37°C in 5% CO_2_ in a McCoy's 5 A Medium supplemented with 10% FBS and antibiotics (10 units/ml penicillin G and 10 *μ*g/ml streptomycin). HT29-MTX cells have been used to study adhesion and invasion of pathogens because of its physiologically relevant characteristics that are responsible for the mucus layer formation.^20, 47^ Caco-2 cells were used as an alternative epithelial cell line to confirm the role of rVvpE in *Muc2* mRNA expression.

### Overexpression and purification of the recombinant elastolytic protease VvpE

To find the functional role of VvpE in HT29-MTX cells, we have previously prepared a recombinant protein of VvpE (rVvpE). Briefly, the coding region of *vvpE* encoding an elastolytic protease was amplified and then subcloned into a His_6_ tag expression vector, pET29a(+) (Novagen, Madison, WI, USA), resulting pKS1202.^19^ The His-tagged VvpE protein was overexpressed in E.coli BL21 (DE3) and then purified by affinity chromatography according to the manufacturer's procedure (Qiagen, Valencia, CA, USA).

### Flow cytometry

HT29-MTX cells were synchronized in the G_0_/G_1_ phase by culture in serum-free media for 24 h before incubation of rVvpE. The necrotic cell death of HT29-MTX cells was detected with an Annexin V and PI staining kit (BD Biosciences) according to the manufacturer's instructions. Briefly, the cells were detached with 0.05% trypsin/EDTA and 1x10^5^ cells were resuspended with Annexin V binding buffer (0.1 M HEPES/ NaOH (pH 7.4), 1.4 M NaCl, 25 mM CaCl_2_). And then the cells were stained with Annexin V (25 *μ*g/ml) and PI (125 ng/ml), and incubated for 15 min at room temperature in the dark. The sample was read by flow cytometry and analyzed using CXP software (Beckman Coulter, Brea, CA, USA).

### Small interfering (si)RNA transfection

Cells were grown until 75% of the surface of the plate and transfected for 24 h with either a siRNAs specific for *ITLN and PKCδ* (GE Dharmacon, Lafayette, CO, USA) or non-targeting (*nt*) siRNA as a negative control (GE Dharmacon) with HiPerFect Transfection Reagent (Qiagen) according to the manufacturer's instructions. The transient knockdown of *ERK1/2* was achieved by transfection with specific amounts of siRNA (100 nM) obtained from Cell Signaling. The siRNA efficacy for *ITLN*, *PKCδ*, and *ERK1/2* were determined by western blot (Supplementary Figure S3).

### RT-PCR

Total RNA was extracted using the RNeasy Plus Mini Kit (Qiagen). Reverse transcription was carried out with 3 *μ*g of RNA using a Maxime RT premix kit (iNtRON Biotechnology, Sungnam, Korea). The cDNAs (5 *μ*l) for mucin isoforms were amplified using the primers described in Supplementary Table S1. The real-time quantifications of pro-inflammatory cytokines and Muc2 were performed using a Rotor-Gene 6000 real-time thermal cycling system (Corbett Research, Mortlake, NSW, Australia) with a QuantiMix SYBR Kit (PhileKorea Technology, Daejeon, Korea) according to the manufacturer's instructions. *β*-actin was used as an endogenous control.

### Mucin (Muc) 2 ELISA

HT29-MTX cells plated on 60-mm culture dishes were grown in FBS-free medium for 24 h and divided into groups according to the experimental protocol. The Muc2 concentration in the culture medium was quantified by an ELISA (Elabscience Biotech, Wuhan, Hubei, China) according to the manufacturer's instructions.

### Western blot analysis and subcellular fractionation

Western blotting was performed as previously described with minor modifications.^48^ The subcellular fractionation method for the isolation of membrane and cytosolic proteins was previously reported.^49^

### Immunoprecipitation

Interaction of ITLN with NOX, NCF1 or caveolin-1 was analyzed by immunoprecipitation and western blotting. Cells were lysed with lysis buffer (1% Triton X-100 in 50 mM Tris–HCl pH 7.4 containing 150 mM NaCl, 5 mM EDTA, 2 mM Na_3_VO_4_, 2.5 mM Na_4_PO_7_, 100 mM NaF, 200 nM microcystin lysine–arginine, and protease inhibitors). Cell lysates (400 *μ*g) were mixed with 10 *μ*g of each antibodies. The samples were incubated for 4 h, mixed with Protein A/G PLUS-agarose immunoprecipitation reagent (Pierce, Rockford, IL, USA) and then incubated for an additional 12 h. The beads were washed four times, and the bound proteins were released from the beads by boiling in SDS-PAGE sample buffer for 5 min. Samples were analyzed by western blotting.

### Detergent-free purification of caveolin-rich membrane fraction

HT29-MTX cells were washed twice with ice-cold phosphate-buffered saline (PBS), scraped into 2 ml of 500 mM sodium carbonate (pH 11.0), transferred to a plastic tube, and homogenized with a Sonicator 250 apparatus (Branson Ultrasonic, Danbury, CT, USA) using three 20 s bursts. The homogenate was adjusted to 45% sucrose by the addition of 2 ml 90% sucrose prepared in 2 (*N*-morpholino) ethanesulfonic acid (MES)-buffered solution consisting of 25 mM MES-buffer solution (pH 6.5) and 0.15 M NaCl and placed at the bottom of an ultracentrifuge tube. A 5%–35% discontinuous sucrose gradient was formed (4 ml each of 5% and 35% sucrose, both in MES-buffer solution containing 250 mM sodium carbonate) and centrifuged at 40 000x*g* for 20 h in a Beckman SW41 Rotor (Beckman Coulter). Twelve fractions were collected and analyzed by 12% SDS-PAGE.

### Intracellular reactive oxygen species detection

2′, 7′-Dichlorofluorescein diacetate (CM-H_2_DCFDA) was used to detect the general ROS production. To quantify the intracellular ROS levels, the cells treated with 10 mM DCF-DA were rinsed twice with ice-cold PBS and then scraped. A 100 *μ*l cell suspension was loaded into a 96-well plate and examined using a luminometer (Victor3; Perkin-Elmer, MA, USA) and a fluorescent plate reader at excitation and emission wavelengths of 485 and 535 nm, respectively.

### Measurement of calcium influx

Changes in intracellular calcium concentrations were monitored using Fluo-3-AM (Invitrogen, Carlsbad, CA, USA) as previously reported with minor modifications.^48^ Fluorescence was excited at 488 nm and the emitted light was observed at 515 nm. All analyses of calcium influx were processed in a single cell, and the results are expressed as the fluorescent intensity.

### Immunofluorescence and immunohistochemical analysis

Either HT29-MTX cells or ileum frozen sections were fixed in 4% paraformaldehyde in PBS for 10 min at room temperature, permeabilized in 0.1% Triton X-100 in PBS for 5 min, and blocked in PBS containing 5% (v/v) normal goat serum (NGS) for 30 min at room temperature. Samples were then stained with primary antibody for overnight at 4 °C. Following three washes with PBS, the samples were incubated with Alexa 488-conjugated goat anti-rabbit/mouse IgM (Invitrogen), and counterstained with PI in PBS containing 5% (v/v) NGS for 2 h. After washing with PBS, samples were mounted on slides and visualized with an Olympus FluoView 300 confocal microscope (Melville, NY, USA) with x400 objective. For immunohistochemical analysis, ileum frozen tissues incubated with primary antibody for overnight at 4 °C were treated with biotinylated secondary antibody solution (Vectastain Elite ABC kit, Vector Laboratories, CA, USA) for 1 h at room temperature. Sections were washed with PBS, incubated in the ABC reagent for 1 h at room temperature, washed again and incubated in a peroxidase solution. Sections were then counterstained with hematoxylin, dehydrated, and coverslipped. Images were acquired using an Axioskop 2 plus microscope equipped with an AxioCam MRc5 CCD camera (Zeiss, Thornwood, NY, USA). Other samples were subjected to hematoxylin and eosin staining for histological examinations.

### Methylation analysis

Genomic DNA from HT29-MTX cells was prepared with the QIAamp DNA Mini Kit (Qiagen, Valencia, CA, USA). The extracted DNA was treated with sodium bisulfite using EzWay DNA Methylation Detection Kit according to the manufacturer's instructions (KOMABIOTECH, Seoul, Korea). The methylation status of *Muc2* gene in HT29-MTX cells was determined by methyl-specific PCR analysis. We conducted methyl-specific PCR of *Muc2* gene promoter at three CPG sites (−289, −274, −193) which have functional role in regulation of *Muc2* expression as described previously.^25^ The primer sequences for each CpG sites of *Muc2* promoter and size of products are summarized in Supplementary Table S2.

### Bacterial strains, plasmids, and culture media

The strains and plasmids used in this study are listed in Table 1. All *V. vulnificus* strains (M06-24/O and M06-24/O *vvpE*) are isogenic and naturally resistant to polymyxin B. Unless otherwise noted, *V. vulnificus* strains were grown in Luria Bertani medium supplemented with 2.0% (wt/vol) NaCl (LBS) at 30 °C. All media components were purchased from Difco (Difco Laboratories, Detroit, MI, USA). *V. vulnificus* were grown to mid-log phase (A_600_=0.500) corresponding to 2 × 10^8^ CFU/ml and centrifuged at 6000 × *g* for 5 min. The pellet was washed with PBS and adjusted to desired colony-forming unit (CFU)/ml based on the A_600_ determined using a UV–VIS spectrophotometer (UV-1800, Kyoto, Shimadzu, Japan) to estimate culture density.

### Mouse models

All animal procedures were performed following the National Institutes of Health Guidelines for the Humane Treatment of Animals, with approval from the Institutional Animal Care and Use Committee of Seoul National University (SNU-140108-4). To determine the mouse lethality, seven-week-old mice (*n*=10) given 250 mg/kg iron dextran were received i.p. inoculation of boiled *V. vulnificus* (Cont), *V. vulnificus* (WT), and a mutant deficient in *vvpE* gene in *V. vulnificus* (M06-24/O *vvpE, vvpE* mutant) (1.2 × 10^2^ CFU/ml) and the survival rate of the mice was recorded for 24 h. To confirm the expression of intestinal Muc2, mice (*n*=5) were received i.g. inoculation of Cont, WT or *vvpE* mutant at 1.1 × 10^9^ CFU/ml for 16 h and sacrificed. We anticipated that 16 h was the minimum duration for colonization activities of the *V. vulnificus* colonization according to the previous our report.^19^ The ileum tissues were embedded in OCT compound and stored at −70 °C. Samples were then cut into 6-*μ*m-thick frozen sections.

### Histologic damage score

Histological parameters were determined in a blinded fashion by two experienced gastrointestinal pathologists as previously described ^50^ with some modification. Briefly, scores were assigned as follows: 0=no damage (normal); 1=slight submucosal and/or lamina propria separation (mild); 2=moderate separation of the submucosa and/or lamina propria and/or edema in the submucosa and muscular layers (moderate); 3=severe separation of the submucosa and/or lamina propria and/or severe edema in the submucosa and muscular layers with regional villous sloughing (severe); or 4=loss of villi and necrosis (necrosis).

### Paneth cells staining

Frozen sections of ileal tissues were stained with phloxine-tartrazine to visualize paneth cells according to the Lendrum reaction. Briefly sections were treated with alum hematoxylin for 1 min, 0.5% phloxine in 0.5% aqueous calcium chloride for 20 min, and then finally differentiated with a saturated solution of tartrazine in 2-ethoxy ethanol (Sigma, St. Louis, MO, USA). With this technique, paneth granules are stained bright red.

### Statistical analysis

Results are expressed as means±standard errors (S.E.). All experiments were analyzed by ANOVA, followed in some cases by a comparison of treatment means with a control using the Bonferroni–Dunn test. Differences were considered statistically significant at *P*<0.05.

## Figures and Tables

**Table 1:** Plasmids and bacterial strains used in this study

**Strain or plasmid**	**Relevant characteristics**	**Reference or source**
*Bacterial strains*		
*V. vulnificus*		
M06-24/O	Clinical isolate; virulent; WT	Laboratory collection
CMM111	MO6-24/O *vvpE*::pKC9844; elastase deficient; *vvpE* mutant	Jeong *et al.*^17^
		
*E. coli*		
BL21 (DE3)	F^-^ *ompT hsdS*_*B*_ (r_B_^-^m_B_^-^) *gal dcm* (DE3)	Laboratory collection
DH5*α*	λ^-^ φ80d*lacZ*ΔM15 Δ(*lacZYA-argF*)*U169 recA1 endA1 hsdR17* (r_K_^-^ m_K_^-^) *supE44 thi-1 gyrA relA1*; plasmid replication	Laboratory collection
S17-1*λpir*	λ-*pir* lysogen; *thi pro hsdR hsdM*^*+*^ *recA* RP4-2 Tc::Mu-Km::Tn7;Tp^r^ Sm^r^; host for *π*-requiring plasmids; conjugal donor	Laboratory collection
		
*Plasmids*		
pET29a(+)	His_6_ tag fusion expression vector; Kn^r^	Novagen
pKS1202	pET29a(+) with 1,827-bp *vvpE*; Kn^r^	Kim *et al.*^19^
pVSV102	*OriV*_R6K*γ*_ *oriT*_RP4_ *oriV*_pES213_ Kn^r^ *gfp*	Dunn *et al.*^51^

Abbreviations: Kn^r^, kanamycin resistant; Sm^r^, streptomycin resistant; Tp^r^, trimethoprim resistant

## Abbreviations

Cont: Control
EDTA: Ethylenediaminetetraacetic acid
EGTA: ethylene glycol tetraacetic acid
ERK: extracellular signal-regulated kinases
FBS: fetal bovine serum
HEPES: 4-(2-hydroxyethyl)-1-piperazineethanesulfonic acid
Ig: immunoglobulin
ITLN: intelectin-1b
JNK: c-Jun N-terminal kinases
MAPK: mitogen-activated protein kinases
M*β*CD: methyl-*β*-cyclodextrin
NAC: N-acetylcysteine
NCF1: neutrophil cytosolic factor 1
NOX2: NADPH oxidase 2
Pan-cad: pan-cadherin
PBS: phosphate-buffered saline
p38: p38 MAPK
PI: propidium iodide
PKC: protein kinase C
PVDF: polyvinylidene fluoride
RFI: relative fluorescence intensity
ROD: relative optical density
ROS: reactive oxygen species
rVvpE: recombinant protein VvpE
SDS-PAGE: sodium dodecyl sulfate-polyacrylamide gel electrophoresis
SE: standard errors
TBST: tris-buffer solution-tween 20
Veh: vehicle
