# Supplementary material for: Vibrio vulnificus VvpE inhibits mucin 2 expression by hypermethylation via lipid raft-mediated ROS signaling in intestinal epithelial cells
Source: Cell Death Dis. 2015 Jun 18;6(6):e1787–. doi: 10.1038/cddis.2015.152 (PMC4669833; doi:10.1038/cddis.2015.152)
Supplement: Supplementary Figure 1 [file cddis2015152x1.docx]

**Supplementary Figure S1. The effect of trypsin in *Muc2* expression.** HT29-MTX cells were incubated with 5, 50 and 500 pg/ml of trypsin for 180 min. Dose responses of trypsin in *Muc2* mRNA expression are shown. *n* = 3.
