# Supplementary material for: Vibrio vulnificus VvpE inhibits mucin 2 expression by hypermethylation via lipid raft-mediated ROS signaling in intestinal epithelial cells
Source: Cell Death Dis. 2015 Jun 18;6(6):e1787–. doi: 10.1038/cddis.2015.152 (PMC4669833; doi:10.1038/cddis.2015.152)
Supplement: Supplementary Figure 2 [file cddis2015152x2.docx]

**Supplementary Figure S2. Effect of rVvpE on the activation of *c*-Src and FAK.** (a) HT29-MTX cells were exposed to rVvpE (50 pg/ml) for 30 min. The effect of rVvpE on the phosphorylation of *c*-Src and FAK was determined by western blot. *n* = 3. (b) Cells were pre-treated with MβCD for 60 min prior to rVvpE (50 pg/ml) exposure for 30 min. Phosphorylation of *c*-Src and FAK was analyzed using western blot. *n* = 3.
