# Supplementary material for: Vibrio vulnificus VvpE inhibits mucin 2 expression by hypermethylation via lipid raft-mediated ROS signaling in intestinal epithelial cells
Source: Cell Death Dis. 2015 Jun 18;6(6):e1787–. doi: 10.1038/cddis.2015.152 (PMC4669833; doi:10.1038/cddis.2015.152)
Supplement: Supplementary Figure 3 [file cddis2015152x3.docx]

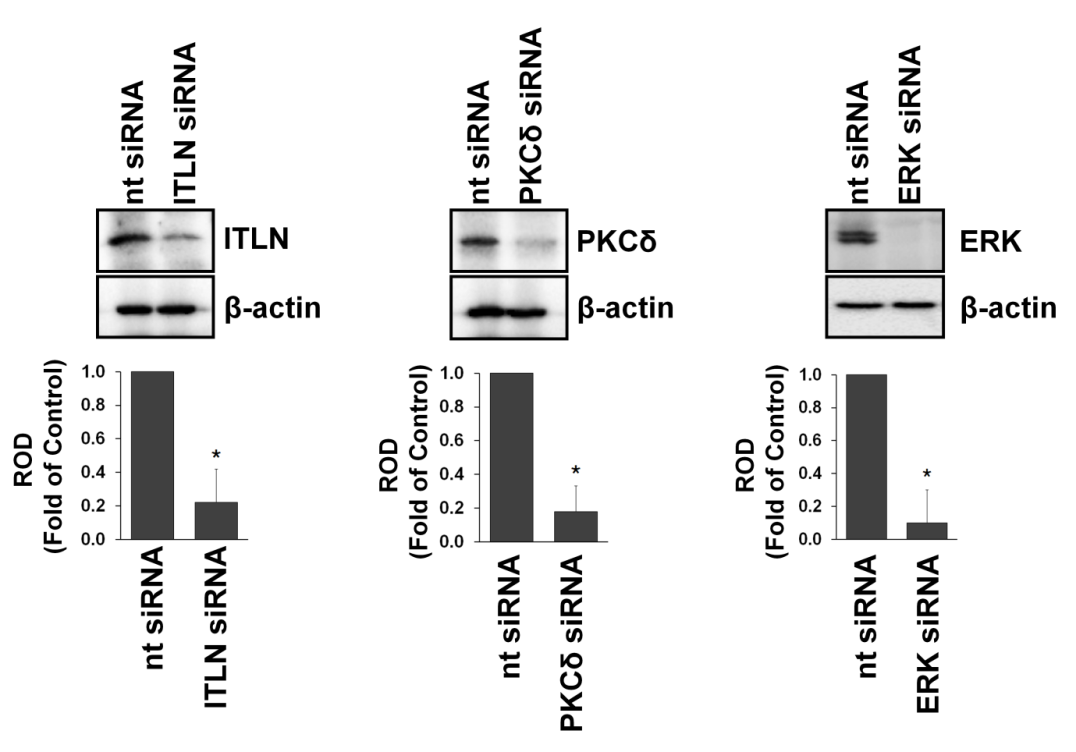


**Supplementary Figure S3. Effect of siRNA on target proteins.** Cells were transfected for 24 h with *ITLN* siRNA (25 nM), *PKCδ* (25 nM), *ERK1/2* (25 nM) or non-targeting (nt) siRNA (25 nM) using HiperFect (Quiagen, Valencia, CA, USA). Expression of ITLN, PKCδ, and ERK1/2 was analyzed using Western blot. The knockdown efficacy of ITLN, PKCδ, and ERK1/2 determined by Western blot was 79%, 82%, and 88%. Error bars represent the means ± S.E. from three independent experiments. *, P < 0.05 vs nt siRNA. Abbreviations: ROD, relative optical density.
