# Supplementary material for: Vibrio vulnificus VvpE inhibits mucin 2 expression by hypermethylation via lipid raft-mediated ROS signaling in intestinal epithelial cells
Source: Cell Death Dis. 2015 Jun 18;6(6):e1787–. doi: 10.1038/cddis.2015.152 (PMC4669833; doi:10.1038/cddis.2015.152)
Supplement: Supplementary Table 1 [file cddis2015152x4.docx]

**Supplementary Table S1. Primers used for polymerase chain reaction**

| **Gene** | **Identification** | **Primer sequence, 5’–3’** | **Size**  **(bp)** |
| --- | --- | --- | --- |
| ***hMUC1*** | Sense  Antisense | CTACCACAGCCCCTAAACCC  AGTAGTCGGTGCTGGGATCT | 165 |
| ***hMUC2*** | Sense  Antisense | CAGCTCATCTCGTCCGTCTC  GCTGGCTGGTTTTCTCCTCT | 298 |
| ***hMUC3*** | Sense  Antisense | GTCAGTGGGATGGCCTCAAA  CCTCAGGGACAGGATCTCCA | 266 |
| ***hMUC4*** | Sense  Antisense | GGAGAAACGCACTTGGTTCG  TCTCCCACCGTCTGTCTTCA | 240 |
| ***hMUC5AC*** | Sense  Antisense | TCTGAGATGCAGGGGTCAGA  GGCTCATTTAAAGCCCGCTG | 290 |
| ***hMUC6*** | Sense  Antisense | ACAACTCCCCCACTTGAGTTC  AGTCGTGGGATGAGTGGACA | 241 |
| ***hβ-Actin*** | Sense  Antisense | AACCGCGAGAAGATGACC  AGCAGCCGTGGCCATCTC | 350 |
