# Supplementary material for: Vibrio vulnificus VvpE inhibits mucin 2 expression by hypermethylation via lipid raft-mediated ROS signaling in intestinal epithelial cells
Source: Cell Death Dis. 2015 Jun 18;6(6):e1787–. doi: 10.1038/cddis.2015.152 (PMC4669833; doi:10.1038/cddis.2015.152)
Supplement: Supplementary Table 2 [file cddis2015152x5.docx]

**Supplementary Table S2. Primers used in Muc2 methylation analysis.**

| **CpG site in Muc2 promoter region** | |  | | **Identification** | | | **Primer sequence, 5’–3’** | **Size**  **(bp)** | |
| --- | --- | --- | --- | --- | --- | --- | --- | --- | --- |
| *-289* | Methylated | | Sense  Antisense | | AGATTTGTTTTTGGTAGGATATTTTTTTTTC  CAACCCTATAACCTAAATACCAAC | | | | 106 |
|  | Unmethylated | | Sense  Antisense | | AGATTTGTTTTTGGTAGGATATTTTTTTTTT  CAACCCTATAACCTAAATACCAAC | | | | 106 |
|  |  | |  | |  | | | |  |
| *-274* | Methylated | | Sense  Antisense | | GTTTATGGYGGGTTAAGGAGTTTGAT  ACCCGAAAAACACATACAACTACTAAAAAAACG | | | | 105 |
|  | Unmethylated | | Sense  Antisense | | GTTTATGGYGGGTTAAGGAGTTTGAT  ACCCAAAAAACACATACAACTACTAAAAAAACA | | | | 105 |
|  |  | |  | |  | | | |  |
| *-193* | Methylated | | Sense  Antisense | | ATAGGGTTGTTTTATTTTGAAGAAGGTTGC  AACATCTACCAAATAATCAAAAAAACAACTA | | | | 137 |
|  |  | |  | |  | | | |  |
|  | Unmethylated | | Sense  Antisense | | ATAGGGTTGTTTTATTTTGAAGAAGGTTGT  AACATCTACCAAATAATCAAAAAAACAACTA | | | | 137 |
|  |  | | |  | |  | | |  |
